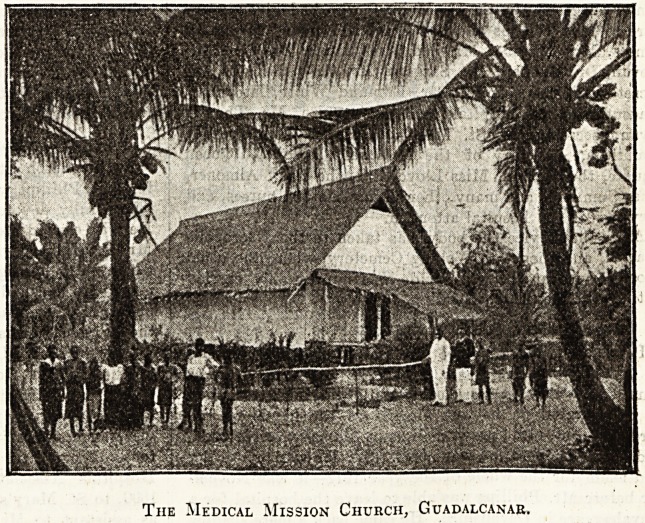# The Welchman Memorial Hospital, Guadalcanar

**Published:** 1914-04-25

**Authors:** 


### THE FIRST HOSPITAL IN THE BRITISH SOLOMON ISLANDS.

### The Welchman Memorial Hospital, Guadalcanar.

BY THE MISSION MEDICAL OFFICER.

On the coast of Guadalcanar, one of the largest of this group of islands, a small hospital has been built, and only just completed, by the Melanesian Mission. Its name records the fact that it has been erected to the memory of the Rev. Henry Welchman, M.R.C.S., for many years priest and doctor to this Mission.

As The Hospital devotes so much of its attention to the construction and equipment of hospitals all over the world, it was thought that some account of the special differences and difficulties in hospital construction and working out coming direct from here might be of interest.

The Welchman Memorial Hospital, Maratovo, Guadalcanar.

### Climate and Construction.

The hospital proper consists of one main building, comprising four wards, dispensary, linen room, and operating theatre, with three sanitary annexes (adjoining the back verandah), one for white patients only, one for male natives, and one for female natives, each containing a bathroom, w.c., and clothes-room, with water laid on from tanks. Two wards, containing three beds in each, with room for one "extra," are provided for white patients; one ward of ten beds for native men, and one of eight beds for native women.

The building is entirely of wood, raised on concrete piles about three feet from the ground.

The roof is ceiled with lining boards underneath corrugated iron, painted with Venetian red, and pierced by three ventilating shafts, one ventilating the two white wards, and one each for the two native wards. The front verandah is ten feet wide, the sides and back six feet.

### Site and Diet.

The site of the hospital is a hill about 100 feet above sea-level, and the land all round has been cleared of bush and trees, and planted with coconuts and sweet potatoes, as well as bananas, yams, sugar-cane, etc., which form a very useful addition to the hospital dietary, where fresh meat, except in the form of poultry, is unobtainable. When the coconuts are about three years old, cows, and possibly sheep, will be imported, and then fresh meat, and, above all, fresh milk, can be obtained regularly.

### Staff Quarters and Gauze Windows.

The Mission steamer *Southern Cross* brought about half the timber, etc., from New Zealand, and the rest had to be obtained from Sydney. The additional buildings are a doctor's house, nurses' home (for two nurses), hospital kitchen and store-room, wash-house, and a small separate building, a little way down the hill, for the treatment of out-patients.

Mosquito-nets are not used; instead, all the windows have wire gauze tacked over them, and the big French window-doors—which are nearly always open—are duplicated by wire-netting doors inside. For water supply we have to depend entirely on rain-water, stored in six 800-gallon tanks.

### Our Patients' Point of View.

The treatment of raw Melanesians in hospital requires an unlimited amount of patience, and plenty of soap and water; their ideas of cleanliness are not ours—*e.g.*, they infinitely prefer to spit on the floor rather than into a cup. The native is accustomed to a hard bed, with a log for a pillow, and doesn't take at all kindly to a mattress—the bed we use here is a smooth wooden stretcher, about six feet by three feet, on two trestles. Its advantages are that the natives feel more at home on it, and it is easily kept clean; its disadvantages are that the patients very easily roll off it, accustomed as they are to rolling

The Medical Mission Church, Guadalcanar.

about on mats on the ground, and also that it is not so firm as an iron bedstead.

After island-sores and "fever," the lungs seem to be the chief source of illness amongst the natives. Pneumonia, bronchitis, and asthma are very common and responsible for many deaths. Filarial disease, with elephantiasis, is also very common; and a baby is not considered properly weaned till it has had yaws, and some of the natives even infect their children with it to "get it over," in spite of the suffering and disfigurement it often causes.

Native Surgery in Theory and Practice.

At present the natives are rather afraid of surgery, but with a judicious selection of cases we hope to get their confidence in time. Their own surgical efforts are rather crude—a bit of broken bottle, or glass, or a sharpened bamboo are used as lancets. In some islands they have an ingenious method of venesection; a tiny arrow tipped with a sharp glass splinter is shot from a very small bow right into or through the vein. Our first operation here was for the removal of dead bone from an enormously hypertrophied tibia, caused about twelve years previously by a boil on the shin being lanced (by a native practitioner) by the simple process of throwing a broken bottle at it—at least that is the story I got!

Appalling deformities result from chronic island sores, with subsequent contraction of tendons, etc.—claw-hands, and toes drawn right up under the sole of the foot are far too commonly seen. Some of these sores heal up rapidly under antiseptic treatment, or simple cleanliness and a light dressing to prevent contamination by flies; others remain unaffected, or go on spreading, in spite of every conceivable form of treatment; and at present the average native much prefers to have his sores dressed daily for months, rather than submit to having them surgically scraped.

An Opportunity of Change.

One great difficulty is the language. In this part of the world there are often many different dialects on the same island, besides separate languages for each island.

Medical histories are very difficult to obtain, and very vague and unreliable. The natives are very independent, and any form of discipline is entirely foreign to their nature.

In conclusion, I can confidently recommend a "locum" in the Solomons to anyone who desires a complete change from medical work in England; he will certainly get it here.

## Figures and Tables

**Figure f1:**
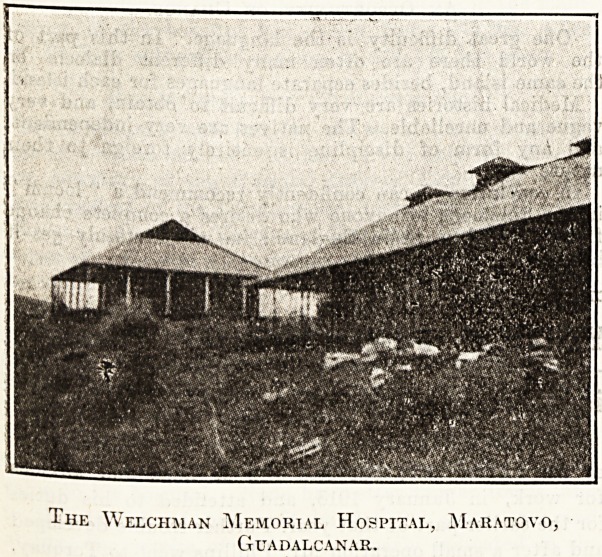


**Figure f2:**